# Specific NFκB subunit activation and kinetics of cytokine induction in adenoviral keratitis

**Published:** 2009-12-25

**Authors:** Jaya Rajaiya, Neda Sadeghi, James Chodosh

**Affiliations:** 1 Department of Ophthalmology, Howe Laboratory, Massachusetts Eye and Ear Infirmary, Harvard Medical School, Boston, MA; 2 University of Oklahoma Health Sciences Center, Oklahoma City, OK

## Abstract

**Purpose:**

Corneal inflammation associated with ocular adenoviral infection is caused by leukocytic infiltration of the subepithelial stroma in response to expression of interleukin-8 (IL-8) and monocyte chemoattractant protein-1 (MCP-1) by infected corneal cells. We have shown that these two chemokines are activated by the mitogen-activated protein kinases (MAPKs) extracellular signal-regulated kinase (ERK) and p38 for IL-8, and Jun-terminal kinase (JNK) for MCP-1. It is also well established that transcription of each of these chemokines is tightly controlled by the nuclear factor kappa B (NFκB) transcription factor family. Therefore, we sought to better understand the differential regulation of chemokine expression by NFκB in adenoviral infection of the cornea.

**Methods:**

Primary keratocytes derived from human donor corneas were treated with signaling inhibitors and small interfering RNA specific to MAPKs, and infected with adenovirus for different time periods before analysis. Activation of specific NFκB subunits was analyzed by western blot, confocal microscopy, electromobility shift assay, and chromatin immunoprecipitation, and chemokine expression was quantified by enzyme-linked immunosorbent assay.

**Results:**

Upon adenoviral infection, NFκB p65, p50, and cREL subunits translocate to the nucleus. This translocation is blocked by inhibitors of specific MAPK signaling pathways. Confocal microscopy showed that inhibitors of the p38, JNK, and ERK pathways differentially inhibited NFκB nuclear translocation, while PP2, an inhibitor of Src family kinases, completely inhibited NFκB nuclear translocation. Western blot analysis revealed that activation of specific NFκB subunits was time dependent following infection. Chromatin immunoprecipitation experiments indicated that binding of NFκB p65 and p50 subunits to the IL-8 promoter upon viral infection was differentially reduced by chemical inhibitors of MAPKs. Electromobility shift assay and luciferase assay analysis revealed that transactivation of IL-8 occurred with binding by the NFκB p65 homodimer or NFκB p65/p50 heterodimer as early as 1 h post infection, whereas MCP-1 expression was dependent upon the NFκB cREL but not the p65 subunit, and occurred 4 h after IL-8 induction. Finally, knockdown of NFκB p65 by short interfering RNA abrogated IL-8 but not MCP-1 expression after adenoviral infection.

**Conclusion:**

The kinetics of NFκB subunit activation are partly responsible for the observed pattern of acute inflammation in the adenoviral-infected cornea. MAPKs differentially regulate chemokine expression in adenoviral keratitis by differential and time-dependent activation of specific NFκB subunits.

## Introduction

An acute inflammatory response to infection or injury occurs in stereotyped stages irrespective of invading organism or mechanism of injury, with neutrophils being the first cells to infiltrate the tissue or body cavity, followed shortly by monocytes [1]. This pattern appears to be the result of the specific induction and activity of chemokines, proteins elicited by cells that induce the directed migration of leukocytes into tissue sites of inflammation [2], by infected or injured cells. Possible molecular mechanisms at play in the tightly controlled pattern of acute inflammation include transcriptional induction, transcriptional repression, and mRNA stability. In particular, it has been shown that AU-rich elements in mRNA contribute stability to the molecule and in part serve to control the kinetics of gene expression of proinflammatory cytokines [3]. Leukocyte infiltration into the corneal stroma represents a critical pathogenic event in viral infection of the cornea. Interleukin-8 (IL-8) is one of the earliest chemokines to be expressed in infection and acts as a first line of defense via its capacity to elicit neutrophil chemotaxis, and to a lesser degree monocyte and T-cell chemotaxis [4-6]. IL-8 induction following viral infection has been shown by many independent research groups [7-10], and a wide variety of cells produce IL-8, including microglia and astrocytes [11-13]. However, in the corneal stroma the molecular mechanisms that regulate IL-8 expression following adenoviral infection remain unclear. Our study focuses on the kinetics of transcription of IL-8 and monocyte chemoattractant protein 1 (MCP-1), another key chemokine in adenoviral keratitis, and on the role of the NFκB transcription factor family in chemokine activation.

The nuclear factor-κB (NFκB) family of transcription factors controls expression of well over one hundred genes, the majority of which participate in regulating innate and adaptive immunity [14,15]. Activation of NFκB occurs within minutes after an appropriate stimulus and leads to strong transcriptional stimulation of both viral and cellular genes [7,16-18]. Analysis of the transcriptional regulation of chemokines induced by viral infection is critical to understanding the pathogenesis of viral keratitis. However, the mechanisms that connect viral infection to chemokine expression by infected stromal cells are poorly understood [7,19-22].

In general, chemokine gene expression is controlled by the NFκB transcription factor family, p65, RELB, cREL, NFκB1 (p50/100), and NFκB2 (p52/105). These proteins form specific homo- or heterodimers for transcriptional activation of target genes in a cell-specific manner. NFκB subunit activation can be achieved through two main pathways: canonical (classical), characterized by the activation of the IκB kinase (IKK) complex, including both IKKα and IKKβ; and non-canonical (non-classical), characterized by activation of NFκB-inducing kinase and IKKα, but not IKKβ [23-28]. Therefore, it is the specific activation of upstream IKKs that represents the point of divergence for NFκB activation. Activation of these pathways has been determined to be both cell and stimulus specific [26-28]. The canonical pathway is the route most commonly activated by pathogens, and is stimulated by pathogen-associated molecular patterns and cytokines. The non-canonical pathway has been described particularly in B lymphocytes, and is stimulated by B-cell activating factor, lymphotoxin β, and CD40L [27,29]. Lipopolysaccharide from *Salmonella enterica* was shown to activate both canonical and non-canonical pathways in primary B cells with activation of both NFκB p50/RELA and p52/RELB heterodimers [26]. Herpes simplex virus type 1 ICP27 protein was shown to activate NFκB via the canonical pathway [30]. While the role of NFκB in apoptosis following adenoviral infection has been explored [31], its role in cytokine regulation due to viral infection has been less fully addressed.

We have earlier shown that NFκB p65 is activated upon adenoviral infection in conjunction with the phosphoinositide 3-kinase/protein kinase B (PI3K/AKT) pathway for cell survival during viral replication [31], but activation of NFκB p65 as a possible mechanism for chemokine induction in adenoviral infection has not been explored. Our prior studies in a mouse model of adenoviral keratitis have shown expression of KC (a homologue of IL-8 in the mouse) within 4 h of infection, followed by MCP-1 at 16 h post infection [32]. In this report we address the role of specific NFκB subunit activation in the kinetics of IL-8 and MCP-1 expression in adenoviral-infected human corneal cells.

## Methods

### Materials

Antibodies to p38, IKKα/β, NFκB p65, NFκB p50, IκB, cREL, and their phosphorylated counterparts were obtained from Cell Signaling Technology (Beverly, MA) and Santa Cruz Biotechnology (Delaware, CA). The anti-human IL-8 and MCP-1 antibodies and biotin-conjugated anti-human IL-8 and MCP-1 antibodies were obtained from BD PharMingen (San Diego, CA). Inhibitors to p38 (SB203580), JNK (SP600125), ERK (PD98059), and Src (PP2) were purchased from Calbiochem (La Jolla, CA). NFκB p65-specific and jumbled control siRNA were purchased from Imgenex (San Diego, CA). The IL-8 luciferase construct was a kind gift from Dr. R. Natarjan (Virginia Commonwealth Univ). The luciferase measuring kit and Renilla constructs were purchased from Promega, (Madison, WI), and the LightShift Chemiluminescent kit from Pierce (Rockford, IL).

### Cell culture and viruses

Primary keratocytes were derived from donor corneas as previously described [33]. Briefly, after mechanical debridement of the corneal epithelium and endothelium, corneas were cut into 2 mm diameter sections, and each section placed in individual wells of 6-well Falcon Tissue Culture Plates (Fisher Scientific, Pittsburgh, PA) with Dulbecco’s Modified Eagle Medium (DMEM), containing 10% heat inactivated fetal bovine serum (FBS), penicillin G sodium, and streptomycin sulfate. Corneal fragments were removed prior to monolayer confluence. Cells were grown and maintained at 37 ^o^C in 5 % CO_2_. Cells from multiple donors were pooled, and the cell monolayers used at passage three. For inhibitor analysis, cells were pretreated with SB203580 (10 µM), SP600125 (25 µM), PD98059 (25 µM), and PP2 (10 µM) at 37 °C for 3 h before infection, and were exposed to the inhibitors at the same concentrations throughout the infection process. The protocol for use of corneas from deceased human donors was approved by the Massachusetts Eye and Ear Infirmary Human Studies Committee, and conformed to the tenets of the Declaration of Helsinki. Human adenovirus species D type 19 (HAdV-D19) used in this study was cultured directly from the cornea of a patient with EKC [33], and purified by cesium chloride gradient. The virus was grown in human lung carcinoma cells (A549 cells, CCL 185; American Type Culture Collection, Rockville, MD) in Minimum Essential Media (MEM) with 2 % FBS, penicillin G sodium, streptomycin sulfate, and amphotericin B. The State of Oklahoma Department of Health confirmed the viral serotype. Typical adenoviral cytopathic effect, positive immunofluorescent staining for adenovirus hexon proteins, and increasing titers of virus within one week after infection of human corneal cells were seen (data not shown).

### Viral infection

Monolayer cell cultures were grown to 95% confluence, serum starved overnight, inhibitor treated for 3 h in Opti-MEM (Invitrogen, Carlsbad, CA), and infected at a multiplicity of infection of 50 or mock infected with virus-free dialysis buffer as a control.

### Transfection

Transient transfections were done in 6 well plates using FuGENE 6 (Roche, Indianapolis, IN) as per the manufacturer’s instructions. A total of 2 μg DNA was transfected, including NFκB p65 or control siRNA (Imgenex, San Diego, CA), IL-8 luciferase construct, and pRL-TK construct, the latter to measure renilla luciferase activity as an internal control. The transfection mixture was prepared by mixing 3 μl of FuGENE 6 in 47 μl of serum-free DMEM, incubated at room temperature for 5 min, the DNA added, and further incubated for 15 min, prior to transfection of 70–80% confluent cells. Viral infections were carried out 48 h post-transfection, and supernatants and cell lysates collected at various time points after infection for enzyme-linked immunosorbent assay (ELISA) and luciferase assay, respectively.

### Immunoblot analysis

Adenoviral- and mock-infected keratocytes were lysed with chilled cell lysis buffer (20 mM Tris, pH 7.4, 150 mM NaCl, 1 mM EDTA, 1 mM EGTA, 1% Triton X-100, 2.5 mM sodium pyrophosphate, 1 mM β-glycerolphosphate, 1 mM Na_3_VO_4_, 1 μg/ml leupeptin, and 1 mM phenylmethylsulphonyl fluoride [PMSF]), and incubated at 4 ºC for 5 min. The cell lysates were cleared by centrifugation at 21,000× g for 15 min. The protein concentration of each supernatant was measured by bicinchoninic acid (BCA) analysis (Pierce, Rockford, IL) and equalized. Twenty micrograms of cell lysates were subsequently separated by 10% sodium dodecyl sulfate polyacrylamide gel electrophoresis (SDS-PAGE) and transferred onto nitrocellulose membranes (BioRad, Hercules, CA). The bands were visualized with an enhanced chemiluminescence kit (Pierce, Rockford, IL).

### Luciferase assay

Short interfering RNA (siRNA) transfected, viral- or mock-infected cells were lysed with 500 μl of 1× partial lysis buffer by rocking at room temperature for 15 min. For the assay, 20 μl cleared cell lysate was added to 100 μl luciferase assay reagent (Promega, Madison, WI), followed by 100 μl Stop and Glo reagent (Promega) to measure renilla luciferase activity as an internal control. IL-8 luciferase activity was then normalized against renilla luciferase activity and analyzed by one-way analysis of variance (ANOVA) with preplanned contrasts, with α=0.05.

### Enzyme-linked immunosorbent assay (ELISA)

The cell supernatants were collected at various time points up to 4 h post infection, and the levels of IL-8 and MCP-1 quantified by sandwich ELISA. The detection limit was 30 pg/ml. Plates were read on a SpectraMax M2 microplate reader (Molecular Devices, Sunnyvale, CA) and analyzed with SOFTmax analysis software (Molecular Devices). The means of triplicate ELISA values for each of the viral- and mock-infected wells were compared by one-way ANOVA with preplanned contrasts, with α=0.05.

### Electrophoretic mobility gel shift assay (EMSA)

Nuclear extracts from adenoviral- or mock-infected keratocytes were prepared using Nucbuster (Novagen, Madison, WI), and binding and supershift assays done using the LightShift Chemiluminescent EMSA kit (Pierce), according to the manufacturers’ instructions. Briefly, chemokine sense and antisense oligonucleotides encoding specific binding sites for NFκB were synthesized (IDT, Coralville, IA). Oligonucleotides were then labeled using Biotin-ddUTP and terminal transferase for 15 min at 37 ^°^C in the labeling buffer and then annealed. For the assay, 10 μg of nuclear extract, labeled oligonucleotide, poly (dI-dC; 1 μg), and poly L-lysine (0.1 μg) were mixed in the binding buffer and incubated at room temperature for 15 min. For comparison, 100 molar excess of unlabelled probe was added to the reactions 15 min before the addition of labeled probe. For the supershift assay, 1 or 2 μg antibody to NFκB p65, p50, or cREL was added to the binding reaction and incubated on ice for 30 min. Protein-DNA complexes were resolved in 5% pre-electrophoresed polyacrylamide gel in 0.5× TBE running buffer and then transferred to a nylon membrane (Roche, Indianapolis, IN). The membrane was then probed for anti-biotin and the bands were detected by chemiluminescence using Kodak films and developed in a QCP X-Ray film processor in which each film is exposed for a preset time. Densitometric analysis of EMSA was performed using ImageQuant 5.2 (Pierce, Rockford, IL) in the linear range of detection, and absolute values then normalized to binding in mock-infected cells. The means of normalized replicate EMSA values for each condition were compared by one-way ANOVA with preplanned contrasts, with α=0.05.

### Confocal microscopy

Keratocytes grown on chamber slides (Nunc, Rochester, NY) were treated with dimethyl sulfoxide or inhibitor for 3 h and then infected with adenovirus or dialysis buffer for 20 min. Cells were partially fixed in 0.05% paraformaldehyde for 10 min, washed in PBS containing 2% FBS, and permeabilized in solution containing 0.1% Triton X-100 for 5 min. After 30 min blocking in 3% FBS-PBS, the cells were incubated in 5 μg/ml of NFκB p65, p50, and cREL primary antibody for 1 h at room temperature, washed, and incubated in Alexafluor-594 and Alexafluor-488 conjugated secondary antibody (Molecular Probes, Eugene, OR) for 1 h more at room temperature. Cells were then washed, fixed in 2% paraformaldehyde, and mounted using Vectashield (Vector labs, Burlingame, CA) mounting medium containing DAPI. Images were taken with an Olympus FluoView 500 confocal microscope using a 60× water immersion objective.

### Chromatin immunoprecipitation (ChIP)

For ChIP, inhibitor treated and viral- or mock-infected cells were subjected to cross-linking for 10 min in 1% formaldehyde at 37 °C, incubated with 0.125 M glycine for 5 min, and then washed twice in cold PBS containing protease inhibitors before resuspension in sodium dodecyl sulfate (SDS) lysis buffer (1% SDS, 10 mM EDTA, 50 mM Tris; pH 8), also containing protease inhibitors. After incubation on ice for 10 min, the suspension was sonicated to reduce the DNA lengths to 200–1,000 bp, centrifuged at 4 °C for 10 min, and the supernatant used for immunoprecipitation. Sonicated extracts were precleared with protein A/G beads (Santa Cruz Biotechnology, Santa Cruz, CA) plus 50 μg salmon sperm DNA for 30 min at 4 °C before incubation with either anti-NFκB p65, p50, cREL or control rabbit IgG overnight at 4 °C. Immunocomplexes were precipitated with blocked protein A/G beads for 1 h. The beads were washed once in low-salt buffer (0.1% SDS, 1% Triton X-100, 2 mM EDTA, 20 mM Tris, 150 mM NaCl; pH 8), once in high-salt buffer (containing 500 mM NaCl), once in LiCl buffer (0.25 M LiCl, 1% NP-40, 1% sodium deoxycholate, 1 mM EDTA, 10 mM Tris; pH 8), and twice in Tris-EDTA buffer. DNA-protein complexes were eluted with 0.1 M NaHCO_3_ and 0.2% SDS, and cross-linking was reversed by incubation at 65 °C for 4 h. Proteinase K (10 μg; Invitrogen, Carlsbad, CA) and 2 μl of DNase-free RNase A (10 mg/ml; Roche) were added for 2 h at 50 °C. The remaining immunoprecipitated DNA was phenol-chloroform purified and subjected to PCR using specific IL-8 and MCP-1 primers. Of the total lysate, 5% was used as a loading control.

## Results

### Activation of NFκB subunits and their inhibitory kinase (IκB) in adenoviral infection

**Figure 1:**
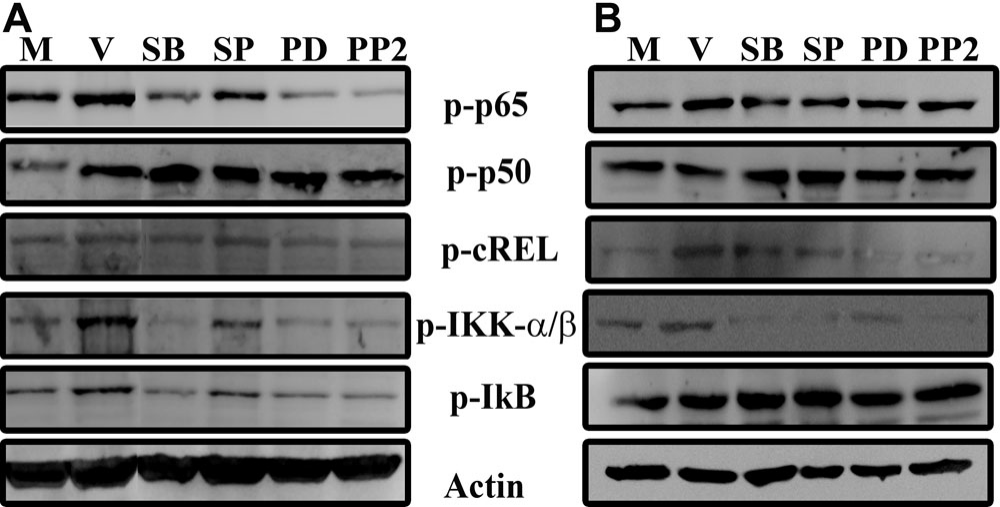
Activation of NFκB subunits upon adenoviral infection in keratocytes is shown. Whole cell extracts from 1 h post viral-infected (V) or mock-treated (M) cells, pretreated with signaling inhibitors SB203580 (SB: p38), SP600125 (SP: JNK), PD98059 (PD: ERK), or PP2 (Src) were run on a 10% sodium dodecyl sulfate polyacrylamide gel electrophoresis and immunoblotted using anti-phospho-NFκB p65, NFκB p50, cREL, IKKα/β and IκB antibodies. Viral-infected cells showed increased activation of NFκB p65, NFκB p50, IKKα/β and IκB both at (**A**) 1 h and (**B**) 4 h after infection. cREL phosphorylation was unchanged at 1 h, but increased at 4 h post infection. Actin levels to determine equivalent protein loading are shown. Phosphorylation of p65, IKKα/β, and IκB was reduced by SB, PD, and PP2 but SP only partially reduced the activation of NFκB p65, IKKα/β, and IκB (**A**). At 4 h post infection, cREL phosphorylation in viral-infected cells was reduced by all inhibitors used (**B**).

In the absence of stimulation, NFκB components are sequestered in the cytoplasm by a tight association with inhibitory proteins of the IκB family. Upon stimulation, IκB is phosphorylated by IKK-containing complexes, releasing NFκB subunits and leading ultimately to the degradation of IκB via the ubiquitin-proteosome pathway [27], and translocation of NFκB to the nucleus for specific transcriptional activity. We infected human keratocytes for 1 and 4 h and performed immunoblot analysis for phosphorylation of specific NFκB subunits, IKKα/β, and IκB. After 1 h, adenoviral infection induced phosphorylation of NFκB p65 and p50, IKKα/β, and IκB but not cREL (Figure 1A). Phosphorylation of p65, IKKα/β, and IκB was reduced by inhibitors of p38 MAPK (SB203580), ERK (PD), and Src (PP2; Figure 1A). However, the JNK inhibitor (SP600125) only partially reduced the activation of NFκB p65, IKKα/β, and IκB, consistent with our previous observation that these MAPK have different downstream targets in adenoviral-infected cells [7,8,34], possibly because JNK is not involved in IL-8 induction. At 4 h post infection, we observed increased cREL phosphorylation in viral-infected cells as compared to mock-treated cells (Figure 1B). These results indicate that IκB and members of the NFκB family are activated rapidly upon adenoviral infection, and that their activation is dependent on upstream kinases shown previously to be important to chemokine expression by adenoviral-infected cells [7,8].

### IL-8 and MCP-1 promoter binding activity of NFκB subunits in adenoviral infection

**Figure 2:**
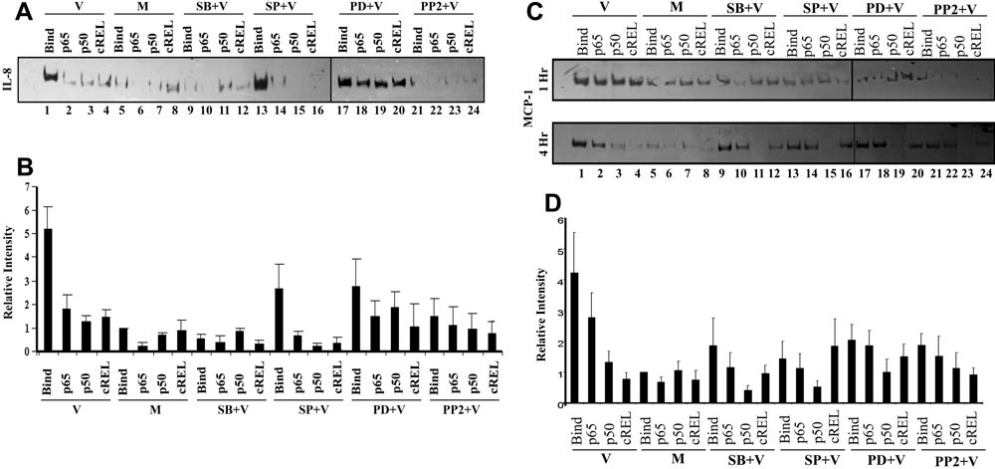
IL-8 promoter binding activity of NFκB subunits is reduced by specific inhibitors. **A**: EMSA was done using 10 μg nuclear extracts after 1 h of viral (V) or mock (M) treatment in cells pretreated with signaling inhibitors SB203580 (SB: p38), SP600125 (SP: JNK), PD98059 (PD: ERK), or PP2 (Src). Binding of p65 to the IL-8 promoter appeared greater in viral-infected cells (lane 1) as compared to mock-treated cells (lane 5), and was reduced in cells pretreated with SB (lane 9) and PP2 (lane 21), but not with SP (lane 13) or PD (lane 17). Supershift assays with NFκB p65, p50 and cREL revealed more shift in viral-infected and SP-treated cells (lanes 2–4 and 14–16), but reduced shift or no shift in mock-treated (lanes 6–8) or other inhibitor-treated cells: SB (lanes 10–12), PD (lanes 18–20), and PP2 (lanes 22–24). **B**: Graphic representation of five independent EMSA experiments. Overall binding was significantly greater in viral-infected cells as compared to mock-treated cells (p<0.0001, ANOVA). IL-8 promoter binding of NFκB subunits was significantly reduced by all four inhibitors (p<0.05). Antibody binding/shift on the IL-8 promoter was not observed in mock-treated cells or in cells treated with any inhibitor prior to infection (p>0.05). **C**: EMSA done for MCP-1 at both 1 and 4 h post infection. Viral infection induced binding/shift relative to mock infection only at 4 h post infection (lanes 1 and 5). Binding at 4 h post infection was reduced in mock-treated and inhibitor-pretreated groups (lanes 5–24). At 4 h post infection, antibody to p50 and cREL reduced binding (lanes 3 and 4), but antibody to p65 (lane 2) did not. **D**: Graphical representation of five independent EMSA experiments for MCP-1 at 4 h post infection. Overall binding was significantly greater in viral-infected cells as compared to mock-treated cells at 4 h post infection (p<0.0001, ANOVA). Binding was reduced in viral-infected cells only by SP (p<0.05). No shift was seen in mock-infected cells due to addition of antibody. In viral-infected cells, a statistically significant shift was seen with p50 and cREL (p<0.05), but not with p65. In viral-infected inhibitor-treated cells only SP reduced binding (p<0.05).

In general, NFκB transcription factors are activated rapidly after exposure to viral infection, resulting in a strong transcriptional regulation of a multitude of early viral and cellular genes [35]. We previously reported the activation of NFκB p65 in a PI3K/AKT dependent manner upon adenoviral infection that ensured delayed cell death and promoted viral replication [31]. EMSA performed on adenoviral-infected keratocytes at 1 h post infection showed NFκB binding to the IL-8 promoter as distinguished from mock infection (Figure 2A). In viral-infected cells, antibody specific to each NFκB subunit appeared to reduce binding, as indicated by reduced density of the primary band, demonstrating the possible involvement of NFκB homodimers or heterodimers in the activation of IL-8 gene expression. Specificity of probe binding was shown by use of 100 molar excess of unlabelled probe (data not shown). We have previously shown that chemical inhibition of JNK has no effect on IL-8 expression by adenoviral-infected keratocytes. Similarly, the JNK inhibitor appeared to have no effect on NFκB binding to the IL-8 promoter, while inhibitors of p38, ERK, and Src reduced NFκB binding (Figure 2A). Statistically, overall binding was significantly greater in viral-infected cells as compared to mock-infected cells (Figure 2B, p<0.0001, ANOVA). IL-8 promoter binding of NFκB subunits was significantly reduced by all four inhibitors (p<0.05). Antibody binding/shift on the IL-8 promoter was not observed in mock-treated cells or in cells treated with any inhibitor prior to infection (Figure 2B, p>0.05). These data correlated with our western blots (Figure 1) and our previous observation that JNK regulates expression of MCP-1 but not IL-8 [34].

At 1 h after infection, we did not observe significant binding of NFκB subunits to the MCP-1 promoter (Figure 2C). However, at 4 h post infection, NFκB subunit binding to the MCP-1 promoter was significantly greater in viral-infected cells as compared to mock-infected cells (p<0.0001). All 3 subunits, but in particular cREL, bound to the MCP-1 promoter at 4 h after infection (Figure 2C), indicating that IL-8 and MCP-1 may be regulated at different time points and by different NFκB dimers. We occasionally but not consistently observed low levels of NFκB p65 binding to the MCP-1 promoter (data not shown). By graphical analysis of multiple EMSA experiments, for MCP-1, only the JNK inhibitor reduced binding, consistent with a JNK-specific activation pathway for MCP-1 (Figure 2D, p<0.05). For the MCP-1 promoter, a binding/shift was seen with p50 and cREL (p<0.05), but not with p65 (p>0.05), suggesting that NFκB p65 does not participate in MCP-1 transcription. In light of our in vivo data showing that KC, an IL-8 homolog, is the first chemokine to be induced upon adenoviral-infection, and that MCP-1 expression is delayed, these results indicate that in adenoviral infection, IL-8 is transactivated by NFκB p65/p65 homodimers and/or NFκB p65/p50 heterodimers, while MCP-1 gene expression is delayed and uses predominantly cREL and NFκB p50.

### Cellular localization of NFκB subunits in adenoviral infection

**Figure 3:**
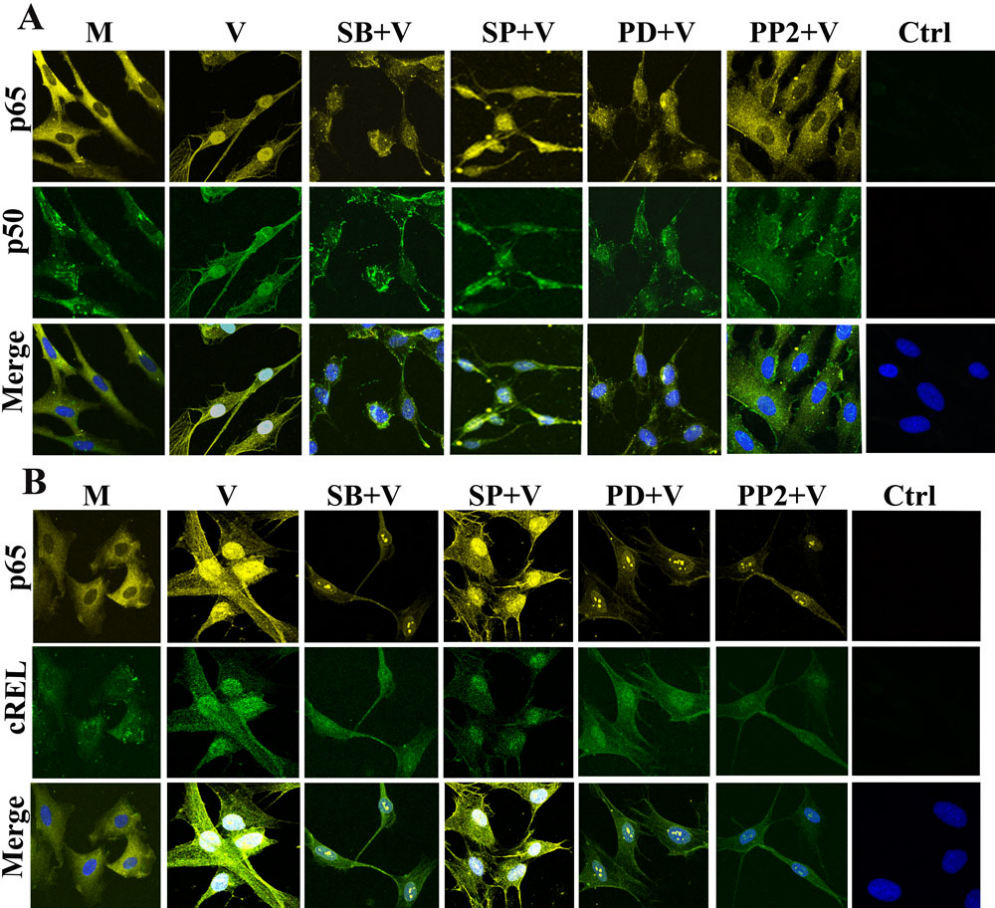
Nuclear translocation of NFκB subunits upon viral infection. Confocal microscopy shows cellular localization of NFκB subunits. **A**: Cytoplasmic localization of NFκB p65 (yellow) is compared to nuclei (blue, DAPI stain) in mock-infected keratocytes (M). Adenoviral-infected (V) keratocytes demonstrate nuclear localization of NFκB p65 and p50. Nuclear translocation of NFκB p65 was reduced in the presence of inhibitors of p38 (SB) and ERK (PD). The nuclear localization of both NFκB p65 and p50 was partially reduced upon pretreatment with an inhibitor of JNK (SP) and completely blocked with an inhibitor of Src (PP2). Isotype control is shown (Ctrl). Viral infection also induced nuclear translocation of cREL (**B**), while all signaling inhibitors tested reduced the nuclear translocation of cREL.

NFκB and IκB shuttle continually between the cytoplasm and nucleus in steady state conditions, resulting in a basal level of NFκB activity [36]. We previously demonstrated increased nuclear localization of NFκB p65 upon adenoviral infection [7]. Now, using confocal microscopy, we confirmed increased nuclear localization of NFκB subunits in viral- but not mock-infected cells (Figure 3A). MAPK inhibitors partially impaired the nuclear localization of NFκB p65, p50, and cREL in viral-infected cells (Figure 3A). Interestingly, SP600125 had less inhibitory effect on nuclear localization compared to other MAPK inhibitors, indicating that the JNK pathway does not mediate NFκB p65 activation. The Src inhibitor PP2 completely suppressed NFκB p65 and p50 nuclear translocation (Figure 3A), suggesting a broad upstream role of Src in these pathways, while downstream MAPKs collaborate in the activation and translocation of NFκB. Confocal microscopy was also performed for the cREL subunit of NFκB. In these experiments, cREL and p65 were both translocated into the nucleus upon viral infection (Figure 3B) as compared to mock-treated cells (Figure 3B). Pretreatment with signaling inhibitors of Src and MAPK reduced both p65 and cREL nuclear translocation, except for SP600125 (Figure 3B).

### Specific NFκB subunits bind to the IL-8 and MCP-1 promoters upon viral infection in a time-dependent fashion

**Figure 4:**
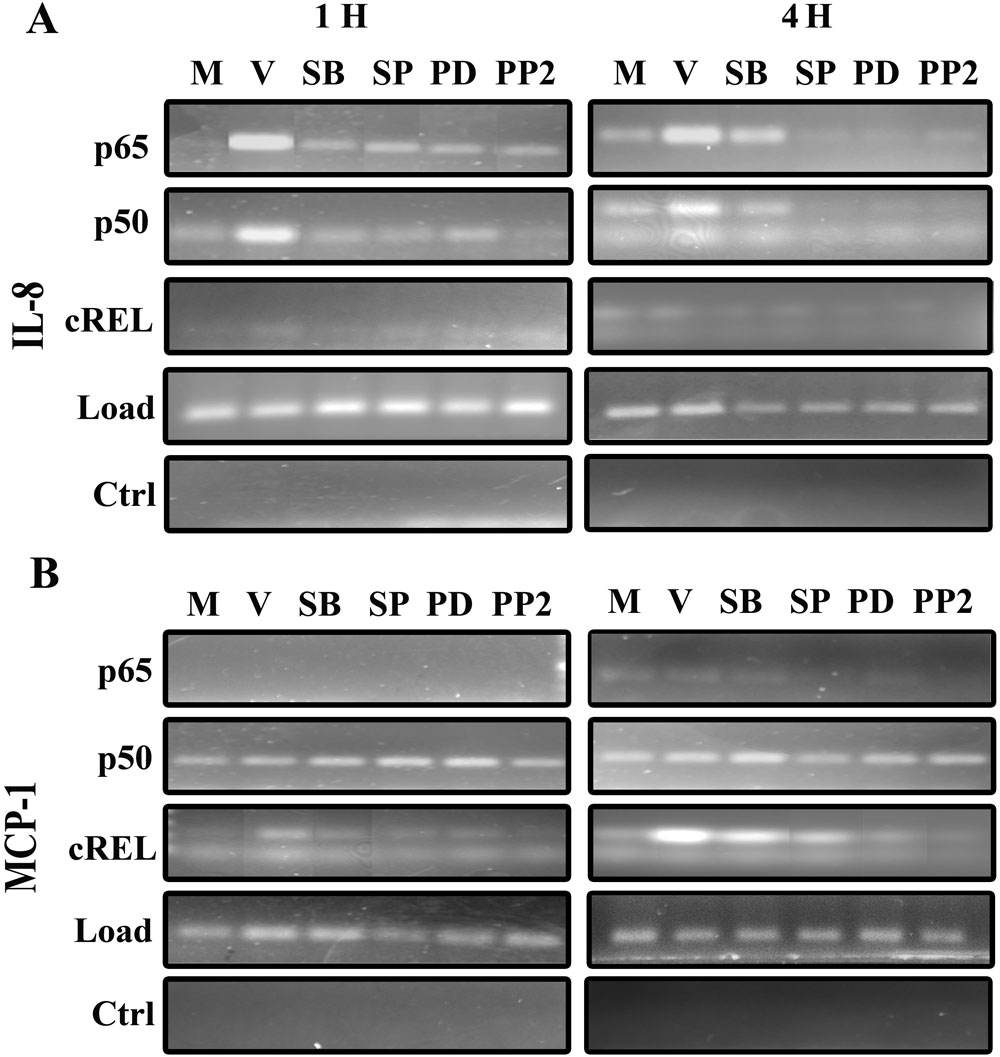
Analysis of promoter binding activity by NFκB subunits using ChIP assay. ChIP was performed with 200 μg lysate. **A**: Binding of NFκB p65 and p50 was observed at both 1 and 4 h post infection on the IL-8 promoter (left and right panels, respectively). A negligible amount of cREL bound to the IL-8 promoter at 1 and 4 h. Inhibitor pretreatment reduced the binding of p65, p50, and cREL on the IL-8 promoter at both time points post infection. For ChIP, 5% of the total lysate was used as a loading control (Load) to show that an equivalent amount of extract was subjected to immunoprecipitation. Isotype control is shown (Ctrl). **B**: p65 binding to the MCP-1 promoter was not observed at 1 or 4 h post infection. p50 bound but showed no apparent change across the pretreatment groups at 1 or 4 h. Binding of cREL on the MCP-1 promoter was apparent at 1 h but increased dramatically at 4 h post infection.

The presence of NFκB in the nucleus is not by itself a direct indication of transcriptional activity [36]. To confirm whether translocated NFκB p65 is transcriptionally active upon viral infection, we analyzed the binding of NFκB p65 and p50 to the IL-8 or MCP-1 promoter using a ChIP technique. Binding of both NFκB p65 and p50 on the IL-8 promoter was considerably increased with adenoviral infection, well above the basal levels seen in mock-treated cells at 1 and 4 h post infection (Figure 4A). As expected, inhibitors of Src and MAPKs dramatically reduced NFκB p65 and p50 binding. Anti-mouse serum control demonstrated the specificity of NFκB p65 and p50 antibody binding, as the control antibodies did not pull down IL-8 DNA. IL-8 promoter binding appeared similar at 1 and 4 h post infection. cREL binding to the IL-8 promoter was negligible. Interestingly, no NFκB p65 was bound to the MCP-1 promoter at 1 or 4 h post infection (Figure 4B). NFκB p50 binding to the MCP-1 promoter was equivalent at both 1 and 4 h after infection and was unaffected by MAPK inhibitors. A marginal increase in cREL binding to the MCP-1 promoter was apparent at 1 h post infection, increasing dramatically at 4 h after infection. cREL binding at 4 h was reduced by all inhibitors utilized.

### NFκ-B p65 is critical for IL-8 induction in adenoviral infection

**Figure 5:**
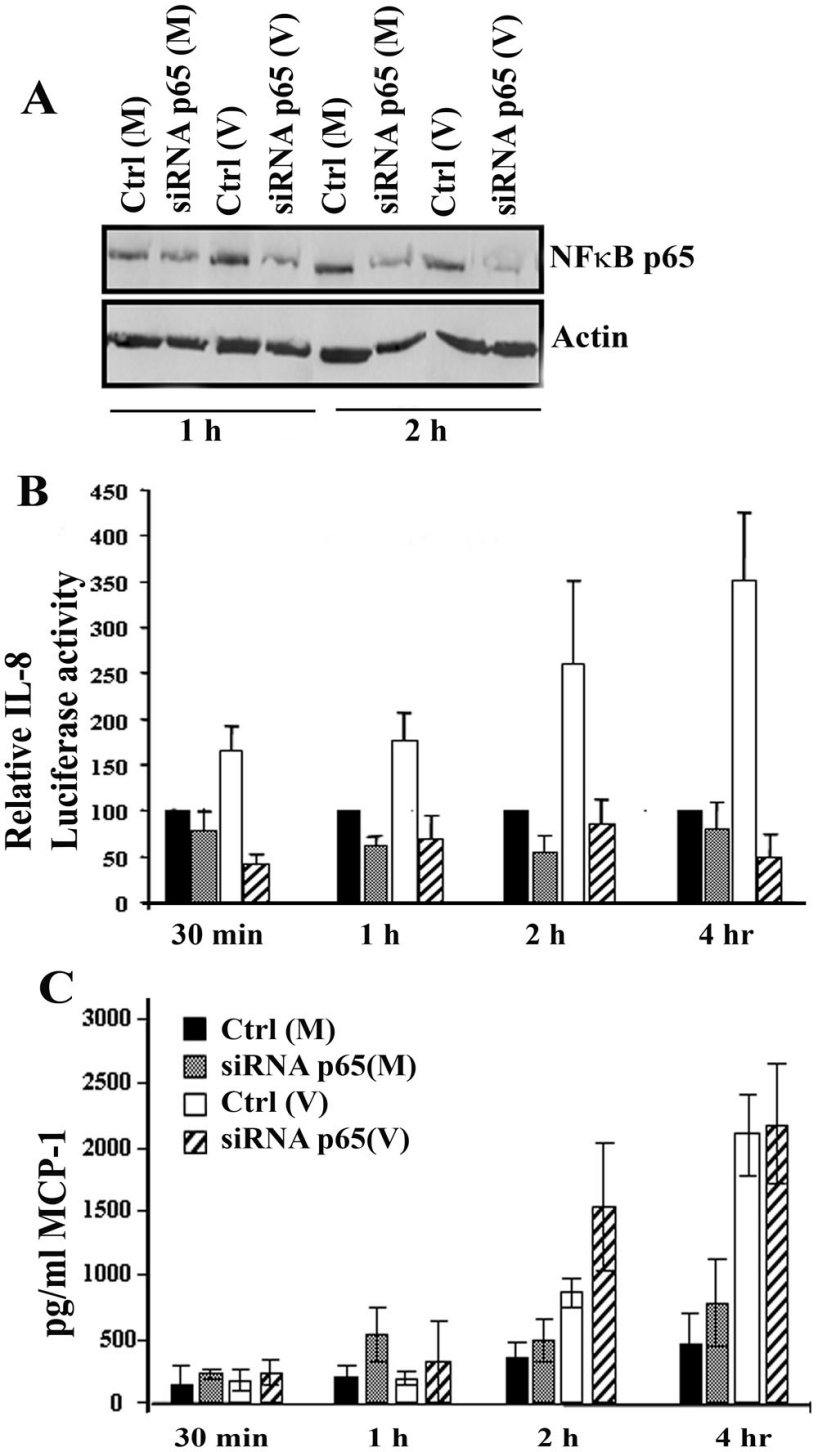
NFκB p65 is indispensable for IL-8 but dispensable for MCP-1 induction. **A**: A representative western blot showed knockdown of NFκB p65. **B**: A luciferase assay demonstrates reduction of IL-8 transcription to mock levels at all times post infection by p65 siRNA treatment (p<0.0001). **C**: MCP-1 ELISA revealed statistically increased expression at 4 h post infection (p<0.0001), but transfected siRNA specific to p65 did not alter MCP-1 expression, as compared to scrambled control siRNA.

Given that adenoviral infection induces NFκB p65 translocation, transcriptional activation, and IL-8 promoter binding, we wished to determine whether p65 is essential to IL-8 induction in adenoviral-infected cells. We used NFκB p65-specific or scrambled control siRNA for these experiments. Figure 5A shows successful knockdown of NFκB p65 expression in keratocytes to 70– 80% in repeat experiments (data not shown). When co-transfected with IL-8 luciferase construct, NFκB p65 specific siRNA reduced IL-8 luciferase activity to basal levels at all time points tested post infection (Figure 5B, p<0.0001), while scrambled control siRNA demonstrated no such effect. Interestingly, MCP-1 ELISA performed on supernatants from the same experiments (Figure 5C) showed no reduction in MCP-1 protein expression in p65 siRNA treated cells, indicating that NFκB p65 is dispensable for MCP-1 induction. MCP-1 expression became significantly elevated in viral-infected cells only at 4 h post infection (p<0.0001). siRNA against p65 did not significantly alter MCP-1 protein expression in viral-infected cells at any time post infection.

## Discussion

The observed difference in the kinetics of IL-8 and MCP-1 chemokine expression after adenoviral infection, in the studies herein, correlates well with the pattern of leukocyte infiltration in vivo [32], but in general, the mechanisms for differential expression of proinflammatory cytokines in infection are not well understood. The stepwise order and timely expression of various inflammatory mediators seem to be preset as a part of a master gene activation program. For example, the consistent differential in time between the expression of interferon gamma and IL-17 during active experimental autoimmune encephalitis in DA rats suggests different roles for these cytokines in the pathogenesis of the disease [37]. Our data is consistent with the idea that the kinetics of cytokine expression in inflammation are due in large part to the interplay between elements that regulate transcriptional induction, transcriptional repression, and perhaps mRNA stability [3].

Two decades after the initial discovery of NFκB, new functions for this ubiquitous transcription factor family continue to emerge. Recent progress in understanding how the immune system senses and responds to pathogens has drawn new attention to NFκB as a key effector of inflammatory responses to infection [27]. Its multiple actions, redundancy in function, and cell specificity have made building a picture of the molecular mechanisms by which NFκB activates cytokine genes in infection a complex undertaking. Viruses that have been previously shown to activate NFκB include human immunodeficiency virus [38], hepatitis B virus [39], hepatitis C virus [40,41], Epstein Barr virus [42], herpes simplex virus [43], and influenza virus [44]. The K13 protein of human herpes virus-8 was shown to mediate IL-8 via NFκB p65, p50 and cREL [10]. Most prior studies of the influence of adenoviral infection on NFκB activation used either recombinant adenoviruses [45], adenovirus vectors [46,47], or isolated adenovirus proteins [7,45,48,49]. The possible roles of NFκB binding kinetics and subunit specificity in chemokine expression by primary keratocytes infected with a native adenovirus have not been previously studied.

Many previous reports demonstrate IL-8 induction through various MAPK pathways converging on NFκB [11,41], and binding of NFκB p65 to the IL-8 promoter [7,50-53]. In our studies, adenoviral-infected cells showed increased nuclear translocation and IL-8 promoter binding for NFκB p65 and p50. Interestingly, we demonstrated by confocal microscopy, EMSA, and western blot that the JNK inhibitor (SP600125) had a minimal effect on NFκB p65 and p50 binding to IL-8 promoter. These data correlate with our earlier reports showing that inhibition of JNK activity in adenoviral-infected cells did not reduce IL-8 expression [34] or inhibit NFκB p65 translocation to the nucleus [7]. However, the ChIP data did appear to show an effect of SP600125 on p65. Also, cREL binding to IL-8 promoter by ChIP assay was negligible, but our supershift assay with cREL on the IL-8 promoter was significant. Further studies will be necessary to resolve these apparent discrepancies.

Our confocal microscopy data demonstrated increased nuclear translocation of NFκB p65, cREL, and p50 in adenoviral-infected cells, whereas no such translocation was observed in mock-treated cells. By confocal microscopy, inhibitors to various MAPKs reduced but did not completely inhibit NFκB subunit translocation to the nucleus, suggesting that NFκB nuclear translocation represents a summation of upstream activity by numerous kinases. Our earlier studies demonstrated that inhibition of Src fully blocked activation of p38 MAPK, ERK, and JNK (32, 35, and 52), suggesting that Src kinases are linearly upstream to all 3 MAPKs. By confocal microscopy, inhibition of Src also effectively blocked NFκB nuclear translocation.

Although IL-8 is one of the best-studied chemokines in host–pathogen interactions, the specific signaling and molecular mechanism underlying the kinetics of its induction remain to be fully elucidated. IL-8 contributes to the chemotaxis of neutrophils but also other leukocytes [11-13], and has become a paradigm chemokine for translational studies of anti-chemokine therapy. Monoclonal antibody to IL-8 was recently utilized successfully as a treatment for localized pustular psoriasis [54]. Our data clearly show that NFκB p65 is critical for IL-8 expression, as knockdown of p65 reduced IL-8 luciferase activity to mock levels at multiple time points post infection. Reduced p65 did not inhibit expression of MCP-1, suggesting again that MCP-1 expression is not NFκB p65 dependent. We suggest that NFκB p65 and cREL play a role in the kinetics of IL-8 and MCP-1 gene regulation, respectively, in adenoviral-infected primary keratocytes. Further studies are needed to clarify the molecular mechanisms underlying MCP-1 induction and how other transcription factors such as Sp1 may regulate chemokine induction in adenoviral infection.
